# Is depression associated with pathways to care and diagnosis delay in people with tuberculosis in Ethiopia?

**DOI:** 10.1017/gmh.2019.17

**Published:** 2019-08-23

**Authors:** F. Ambaw, R. Mayston, C. Hanlon, A. Alem

**Affiliations:** 1 School of Public Health, Bahir Dar University, Bahir Dar, Ethiopia; 2 Department of Psychiatry, College of Health Sciences, School of Medicine, Addis Ababa University, Addis Ababa, Ethiopia; 3 King's College London, Institute of Psychiatry, Psychology and Neuroscience, Centre for Global Mental Health, London, UK; 4 Centre for Innovative Drug Development and Therapeutic Trials for Africa (CDT-Africa), College of Health Sciences, Addis Ababa University, Addis Ababa, Ethiopia

**Keywords:** Depression, diagnosis-delay, Ethiopia, pathways-to-care, tuberculosis

## Abstract

**Background.:**

Co-morbid depression is common in people with tuberculosis (TB). Symptoms of depression (low energy, impaired concentration, decreased motivation and hopelessness) may affect help-seeking; however, this impact has not been studied so far. The objectives of this study were to assess the impact of co-morbid depression on diagnostic delay, pathways to care, and to identify if it mediates other factors associated with diagnostic delay.

**Methods.:**

We analyzed cross-sectional data collected from 592 adults with newly diagnosed TB. We assessed probable depression using Patient Health Questionnaire, nine items (PHQ-9) at a cut-off 10. Data on diagnosis delay, pathways to TB care, socio-demographic variables, stigma, types of TB, substance use, co-morbid chronic illnesses, and perception about TB were assessed using a structured questionnaire. Generalized structural equation modelling was used to analyze the data.

**Results.:**

A total of 313 (52.9%) participants had probable depression. Pathway to TB care was direct for 512 (86.5%) of the participants and indirect for 80 (13.5%) of them. The median diagnosis delay was 12.0 weeks. Depression did not have a statistically significant association with pathways to TB care (*β* = −0.45; 95% CI−1.85 to 0.96) or diagnostic delay [adjusted odds ratio (AOR) = 0.90; 0.77–1.06]. Indirect pathway to TB care was positively associated with diagnosis delay (AOR = 2.72; 95% CI 1.25–5.91).

**Conclusions.:**

People with TB who had co-morbid probable depression visited the modern health care as directly as and as soon as those without co-morbid depression. How socio-demographic factors influence pathways to care and diagnosis delay require qualitative exploration.

## Introduction

Tuberculosis (TB) is one of the leading causes of death from an infectious disease in the world (World Health Organization, 2018) and the third highest contributor to disability adjusted life years (DALYS) in Ethiopia (GBD 2016 DALYS and HALE Collaborators, 2017). Timely diagnosis and correct treatment cure most people who develop TB (World Health Organization, 2016). Delayed diagnosis leads to more severe clinical presentations (Virenfeldt *et al*., 2014), longer duration of infectivity and poorer prognosis. Rapid diagnosis is essential in order to decrease the incidence rate of TB (Friedland, 2011) to the extent required to meet the Global End-TB target by the year 2030 (10% decrease/year) (World Health Organization, 2015). Delayed diagnosis of TB has been found to be associated with HIV status, residence, geographical or socio-psychological barriers, health care provider contacted upon seeing symptoms, type of TB, age, sex, substance use, level of education, and stigma (Storla *et al*., 2008). Many of these variables are known to be associated with depression in people with TB (Ambaw *et al*., 2017); but the impact of co-morbid depression on diagnostic delay has not been investigated (Storla *et al*., 2008).

Co-morbid depression, a common disorder in people with TB (Trenton & Currier, 2001; Doherty *et al*., 2013; Sweetland *et al*., 2014; Sweetland *et al*., 2017, 2018), may negatively affect timely diagnosis and service utilization (Sweetland *et al*., 2018). People with depression are known to have poor service utilization even in high-income settings (Andrews *et al*., 2001; Oliver *et al*., 2005). Depression is associated with reduced energy levels, impaired concentration and memory, low motivation and hopelessness (Katon, 2011). Co-morbid depression could also obscure recognition of TB symptoms because of the overlap of symptoms of depression with physical illnesses (Pachi *et al*., 2013) and predominantly vague somatic presentation of depression (Keely *et al*., 2004). Impaired uptake of health education messages may also lead to more indirect pathways to TB care (e.g. via traditional and religious healing), which in turn has been shown to result in diagnostic delay (Finnie *et al*., 2011).

The aim of this study was to assess the association between depression and diagnosis delay, pathways to TB care, and its role in mediating the association between diagnosis delay and the known risk factors in people with TB. Our hypotheses were as follows:

1. Co-morbid depression will be associated with delayed diagnosis in people with TB and may be a mediator of the effects of known risk factors for diagnostic delay.
2. Co-morbid depression in people with TB will interfere with effective help-seeking and greater use of indirect pathways to care.

## Methods

### Design

We analyzed cross-sectional data focusing on time to TB diagnosis, pathways to TB care, and depression from the baseline of our cohort study investigating the impact of co-morbid depression on TB outcomes in primary care settings in Ethiopia. In that cohort study, adults with newly diagnosed TB were recruited at the time of initiating Directly Observed Treatment Short-Course (DOTS) and followed up to the end of treatment (6 months after initiation). Data were collected at three time-points: baseline (before anti-TB treatment), at 2 months (end of intensive phase) and at 6 months (end of continuation phase). The exposure variable was depression. The primary outcome variable was TB treatment outcomes (treatment complete, cure, treatment failure, treatment default, or death). Secondary outcome variables were quality of life, disability, and pathways to TB care. Control variables included socio-demographic variables, substance use, perceived social support, perceived cause of TB, perceived barriers to modern TB care, stigma related to TB, and perceived severity of TB (Ambaw *et al*., 2015).

### Study setting

The study was conducted from December 2014 to July 2016 in 14 primary care centres in South central Ethiopia (the Silti and Gurage zones) and northern Ethiopia (Bahir Dar zone). Two of the primary care centres were hospitals and 12 were health centres. Similar TB services were provided at the different types of facility: providers of TB care were nurses or public health officers, the same treatment guidelines and medications were used, and only outpatients were included. The frontline nurses and public health officers working in those primary care centres had received training in the management of mental disorders according to the evidence-based WHO Mental health gap action programme intervention guide (mhGAP-IG) for mental, neurological and substance use disorders in non-specialized health care settings (WHO, 2016).

### Eligibility criteria

- People attending the selected health centres for TB treatment who were within 1 month of starting anti-TB treatment
- Aged 18 years and above
- Did not have plan to move out of the study area
- Not too ill to be interviewed at baseline as perceived by the interviewer or the prospective participant
- Were not admitted to in-patient unit for more than 5 days in the last 1 month as the additional stressors of being hospitalized represent a different range of risk factors for depression.
- Did not have Multidrug-Resistant Tuberculosis (MDR-TB); people with MDR-TB constitute a different population because their treatment and outcomes are different (more toxic medications for a much longer duration and poorer prognosis) and MDR-TB is a more feared and stigmatized condition (Vega *et al*., 2004). Furthermore, only one of the study health facilities had recently started a service for people with MDR-TB.
- Were not on re-treatment for TB as people who experienced previous treatment failures are at high risk of MDR-TB and constitute a different risk group for depression.

### Sample size determination

The sample size was based on the primary objective of the longitudinal study which was to examine the effect of depression on default from anti-TB treatment (Ambaw *et al*., 2015). The following parameters were used in that calculation: 80% power, 95% confidence level, 2.5% prevalence of treatment default among patients with TB and without depression, 7.5% prevalence of treatment default among people with TB and co-morbid depression, a ratio of 2:1 of non-exposed (not depressed) to exposed (depressed) participants. This provided a required sample size of 639. With a contingency of 10% (64) for possible loss to follow up, the target sample was 703 people with TB. However, only 657 people with newly diagnosed TB were recruited into the study because of the scarcity of cases that meet our selection criteria (Ambaw *et al*., 2017). Because one of the required variables, stigma, was measured at 2 months of follow up, 65 (25 transferred out of study area, 22 died, nine diagnoses changed, six lost to follow up, and three refused to participate in the second month) of the 657 participants did not have value for it (Ambaw *et al*., 2018). Therefore, we analyzed data from the remaining 592 participants who had complete responses for the variables of interest for this study.

### Recruitment and ethics

People with newly diagnosed TB who fulfilled the inclusion criteria were identified, informed and invited to participate in the study by health professionals running TB clinics at the health facilities. When the individuals expressed interest to participate, they were linked to trained nurse research assistants who provided them detail information, sought written informed consent or witnessed thumb print, and carried out the data collection generally at the health facilities. The information provided to participants was explained face-to-face and delivered in written form to participants. The proposal was approved by the Institutional Review Board of College of Health Sciences of Addis Ababa University (number 027/14/Psy) before data collection. In the process of data collection, respondents who endorsed the suicide item of PHQ-9 were referred to health workers within the health facilities for further evaluation and treatment.

### Dependent variables

#### Diagnostic delay

Diagnostic delay was defined as the time interval between the onset of TB symptoms and diagnosis of TB (Belay *et al*., 2012). Participants were asked ‘how long did your TB symptoms last before your illness was diagnosed as TB?’ In this study, the diagnosis was considered to be ‘delayed’ if the interval between the onset of symptoms and the date of diagnosis was above 12 weeks. Because there was no standard definition for diagnosis delay, the median of the sample scores (12 weeks) was used as a cut off value to operationally define the variable. A systematic review had found out that previous quantitative studies commonly used the median split to define delay (Finnie *et al*., 2011).

#### Pathways to TB care

This was measured using a modified version of the World Health Organization (WHO) encounter form for mental disorders (WHO, 1987). Pathway to TB care was categorized as ‘direct’ if people with TB directly contacted the modern health care system where TB symptoms were recognized for the current illness episode. Pathways were categorized as ‘indirect’ if participants indicated that they had sought help from the traditional health care system prior to attending biomedical services.

### Independent variables

#### Probable depression:

Depression was an independent variable hypothesized to be associated with both pathways to TB care and diagnosis delay. It was also considered as a mediator of the relationship between other independent variables and the dependent variables. Probable depression was measured using the nine-item version of the Patient Health Questionnaire (PHQ-9). Globally, the scale has been widely used in surveys, effectiveness trials and cohort studies (Kroenke *et al*., 2010). In Ethiopia, it has been validated in two settings and was found to be useful in screening depression in adult outpatients (Gelaye *et al*., 2013; Hanlon *et al*., 2015). The optimum cut-off point was five and above in primary healthcare centres in a rural district (Hanlon *et al*., 2015) and ten and above in outpatient medical clinics in a referral hospital in Addis Ababa (Gelaye *et al*., 2013). We applied the more conservative cut-off point of ten and above to define probable depression given the potential overlap between TB and depression symptoms. The baseline data of this study indicated that the PHQ-9 had a single-dimensional structure, Cronbach's alpha of 0.81, and a mean inter-item correlation coefficient of 0.33 (Ambaw *et al*., 2017). In this paper, the term depression is used instead of probable depression for simplicity.

#### Socio-demographic variables

Age, sex, marital status, level of education, religion, household income, occupation and place of residence (urban *v.* rural) were measured by self-report. Household income was measured by asking the participants to estimate the monthly total income of their household. When the participant was a farmer, we changed the estimates of annual income in kind to cash using the local market price. We converted the monthly income into annual income.

#### Substance use

Alcohol, tobacco and khat use were measured using the WHO Alcohol, Smoking and Substance Involvement Screening Test (ASSIST) (version 3.1) (WHO, 2010a). ASSIST was designed for use across different cultural settings. The instrument's psychometric properties have been tested using data from multiple countries, including low, middle, and high-income countries and shown to be valid, reliable and easy to administer across settings (Humeniuk*et al*., 2008).

*Perceived social support*: Perceived social support was measured using the three-item Oslo Scale of Perceived Social Support with scores ranging from 3 to 14 (Meltzer, 2003). The scale was previously reported to work well in Ethiopia in TB patients (Duko *et al*., 2015). The total score was categorized in tertiles which were then operationalized as poor, moderate and strong perceived social support.

#### Co-morbid illness

Data on the presence of chronic illnesses other than TB were obtained by asking the question ‘Have you ever been told by health professionals that you have cardiac illness, hypertension, diabetes mellitus, depression or mental illnesses other than depression?’ HIV status was recorded from the TB register.

#### Type of TB (pulmonary or extra-pulmonary)

Information extracted from the TB register in the health facilities.

#### Disability

Disability was measured using the interviewer administered version of the 12-item World Health Organization Disability Assessment Schedule, version 2.0 (WHODAS 2.0) (WHO, 2010b). Studies have demonstrated the usefulness of the tool for assessing disability in primary care patients with depression (Chwastiak & Von Korff, 2003). The scale has been previously used in Ethiopia (Mogga *et al*., 2006; Senturk *et al*., 2012; Habtamu*et al*., 2017). Scores were divided into tertiles and operationalized as no or low disability, moderate and severe disability.

#### Perceptions about TB

Perceived severity of TB, perceived benefits of TB treatment, perceived barriers to modern TB treatment and perceived causes of TB were captured by asking the respondents, ‘How severe do you think TB is (mild, moderate or severe), how helpful do you think medications for TB are (not helpful, somehow helpful or very helpful), and do you have barriers to take your medications as prescribed (yes or no), what do you think is the cause of TB?’, respectively.

#### TB-related stigma

TB-related stigma was measured at the second assessment using a 10-item TB stigma scale adapted from Macq *et al*. (2006), translated into Amharic, and piloted (Ambaw *et al*., 2015). In this sample, the scale had an alpha value of 0.84 and a mean inter-item correlation coefficient of 0.34. Scores were cut into three equal parts and operationalized as low, moderate, and high stigma.

### Analyses

Data were analyzed using STATA version 15 (StataCorp. URL: http://www.stata.com). Descriptive statistics were used to summarize variables and to describe participants. Logistic regression was fitted using generalized structural equation modelling (GSEM) to identify the association between depression and the dependent variables (diagnostic delay and pathways to TB care) as well as the role of depression as a mediator between other independent variables and the dependent variables. The role of pathways to TB care as a mediator between diagnostic delay and all other independent variables including depression was assessed the same way. GSEM allows modelling categorical outcomes and generalized responses without the need to fulfil the requirement of multivariate normality (Rabe-Hesketh *et al*., 2004; StataCorp, 2017). The inclusion of independent variables in the multivariable analysis was based on its theoretical importance and adequacy of the number of participants in cells for each category (Tabachnic & Fidell, 2007). Because the study sites were located in two regions of the country, clustered robust standard errors were used. *p* values <0.05 were considered statistically significant. Associations have been reported using odds ratios and beta (*β*) coefficients depending on the availability of options for GSEM in STATA 15. The Strengthening the Reporting of Observational Studies in Epidemiology (STROBE) guidelines have been used to report our findings (von Elm *et al*., 2007).

## Results

### Socio-demographic characteristics of the participants

Just over half of the participants (*n*  =  312; 52.7%) were male, 444 (75.0%) had primary education or no formal education and the median age of the participants was 30 years with range 18–85 years (Table 1).

**Table 1.** Socio-demographic characteristics of participants (*n*  =  592)

Variable	Number (%)
Sex	
Male	312 (52.7)
Female	280 (47.3)
Age in years (mean = 33.8 ± 14; median = 30)	
18–34	364 (61.5)
35–49	135 (22.8)
50–85	93 (15.7)
Marital status	
Single	192 (32.4)
Married	329 (55.6)
Widowed or divorced	71 (12.0)
Level of education	
No formal education	209 (35.3)
Primary education	235 (39.7)
Secondary or above	148 (25.0)
Occupation	
Unemployed	35 (5.9)
Government employee	52 (8.8)
Self-employed	118 (19.9)
Farmer	159 (26.9)
Student	37 (6.3)
Housewife	105 (17.7)
Daily labourer	42 (7.1)
Other	44 (7.4)
Annual household income (median = 9366.00 Birr)	
Below median	298 (50.3)
Median or above	294 (49.7)
Religion	
Christian	387 (65.4)
Muslim	205 (34.6)
Residence	
Urban	327 (55.2)
Rural	265 (44.8)
Ethnicity	
Amhara	275 (46.5)
Gurage	176 (29.7)
Mareko	64 (10.8)
Silte	62 (10.5)
Oromo	9 (1.5)
Other	6 (1.0)
Perceived social support	
Poor	244 (41.2)
Moderate	193 (32.6)
Strong	155 (26.2)
TB stigma score	
Low	177 (29.9)
Moderate	232 (39.2)
High	183 (30.9)

### Illnesses and substance use characteristics

Just over half of the participants (*n*  =  313; 52.9%) had probable depression. In total 445 (75.3%) perceived that TB is a severe illness and only 82 (13.9%) perceived that TB was caused by microorganisms. A total of 62 (10.2%) were living with HIV. Moderate to high risk of alcohol, khat and tobacco was reported by 74 (12.5%), 96 (16.2%), and 29 (4.9%) of participants, respectively (Table 2).

**Table 2.** Illness, substance use and pathways to TB care (*N*  =  592)

Variables	Number (%)
Diagnosis delay in weeks (median = 12; range = 0.3–520)	
12 weeks or less	345 (58.3)
Above 12 weeks	247 (41.7)
Pathways to TB care	
Direct (modern care)	512 (86.5)
Indirect (traditional care)	80 (13.5)
Probable depression	
No	279 (47.1)
Yes	313 (52.9)
Type of TB	
Pulmonary	340 (57.4)
Extra pulmonary	252 (42.6)
Perceived severity of TB	
Mild to moderate	147 (24.8)
Severe	445 (75.2)
Perceived barriers to modern care	
No	458 (77.4)
Yes	134 (22.6)
Perceived micro organism as a cause of TB	
No	510 (86.2)
Yes	82 (13.9)
Perceived benefit of modern TB care	
Not helpful	2 (0.30)
Somehow helpful	23 (3.9)
Helpful	567 (95.8)
HIV status	
Negative	455 (76.9)
Positive	62 (10.5)
Unknown	75 (12.7)
Level of disability	
Low or no disability	177 (29.9)
Moderate disability	231 (39.0)
High disability	184 (31.1)
Hypertension	
No	591 (99.8)
Yes	1 (0.2)
Cardiac illness	
No	590 (99.7)
Yes	2 (0.3)
Diabetes mellitus	
No	587 (99.2)
Yes	5 (0.8)
Prior depression	
No	592 (100)
Yes	0
Mental illness other than depression	
No	592 (100.0)
Yes	0
Level of alcohol risk	
Low	518 (87.5)
Moderate	66 (11.1)
High	8 (1.4)
Level of tobacco risk	
Low	563 (95.1)
Moderate	27 (4.6)
High	2 (0.3)
Level of khat risk	
Low	496 (83.8)
Moderate	87 (14.7)
High	9 (1.5)

### Diagnostic delay and pathways to TB care

The median delay to diagnosis was 12 weeks (range 0.3–520.0 weeks). A total of 512 (86.5%) participants contacted the modern health care system directly. The remaining 80 (13.5%) participants visited the traditional health care system before contacting the modern health care system where they had their illness diagnosed as TB (Table 2).

### Factors associated with pathways to TB care and diagnostic delay

There was no association between depression and diagnostic delay (Adjusted odds ratio (AOR)  =  0.90; 0.77, 1.06) or with pathways to TB care (adjusted *β* = −0.45; 95% CI−1.85 to 0.96). Indirect pathways to TB care (AOR = 2.72; 95% CI 1.25–5.91), male gender (AOR = 1.62; 95% CI 1.39–2.02), absence of formal education (AOR = 3.01; 95% CI 2.25–4.03), being a rural resident (AOR = 1.67; 95% CI 1.49–1.88), being Muslim (AOR = 1.57; 95% CI 1.29–1.92), having moderate (AOR = 2.11; 95% CI 1.03–4.29) or severe disability (AOR = 1.52; 95% CI 1.35–1.71), having extra-pulmonary TB (AOR =  1.46; 95% CI 1.07–2.00), having higher household income (AOR =  1.39; 95% CI 1.15–1.67), and moderate- to- high risk of alcohol use (AOR = 1.34; 95% CI 1.14–1.57) were associated directly with delay of diagnosis in people with TB. HIV positive status (AOR =  0.81; 95% CI 0.67–0.99) and age group 50–85 years (AOR =  0.82; 95% CI 0.75–0.88) had inverse relation with delaying diagnosis of TB.

Absence of formal education (adjusted *β* =  0.41; 95% CI 0.14–0.67) or having just primary education (adjusted *β* =  0.78; 95% CI 0.58–0.97), perceived social support (adjusted *β* = 0.36; 0.20–0.53), severe disability (adjusted *β* =  1.86; 95% CI 1.68–2.05), higher household income (adjusted *β* =  0.31; 95% CI 0.24–0.38), and moderate- to- high risk of khat use (adjusted *β* =  1.12; 95% CI 0.92–1.31) were positively associated with diagnostic delay through increasing visits to the traditional health care system before reaching modern care facilities. On the other hand, rural residence (adjusted *β* = −0.33; 95% CI −0.44 to −0.23), unknown HIV status (adjusted *β* = −0.87; 95% CI −1.10 to −0.65), and perceiving TB as a severe illness [adjusted *β* = −0.93 (−1.21 to −0.65)] indirectly decreased diagnostic delay through decreasing visits to the traditional health care system (Table 3). The path diagram of the analysis is shown in online Supplementary Figure S1).

**Table 3.** Factors associated with pathways to TB care and diagnosis delay (*n* =  592)

Variables	Delay status		Diagnosis delay AOR (95% CI)	Pathways to care Adjusted *β* (95% CI)
	No delay Number (%)	Delay Number (%)		
Pathways to TB care				
Direct (modern)	311 (60.7)	201 (39.3)	1	
Indirect (traditional)	34 (42.5)	46 (57.5)	2.72 (1.25–5.91)	
Presence of probable depression:				
No	168 (60.2)	111 (39.8)	1	1
Yes	177 (56.6)	136 (43.5)	0.90 (0.77–1.06)	−0.45 (−1.85 to 0.96)
Sex				
Male	180 (57.7)	132 (42.3)	1.62 (1.39–2.02)	1
Female	165 (58.9)	115 (41.1)	1	0.21 (−0.42 to 0.84)
Age in years				
18–34	226 (62.1)	138 (37.9)	1	1
35–49	70 (51.9)	65 (48.1)	1.14 (0.80–1.62)	−0.05 (−0.83 to 0.74)
50–85	49 (52.7)	44 (47.3)	0.82 (0.75–0.88)	−0.40 (−1.15 to 0.34)
Level of education				
No formal education	95 (45.5)	114 (54.5)	3.01 (2.25–4.03)	0.41 (0.14–0.67)
Primary education	147 (62.6)	88 (37.4)	1.17 (0.77–1.77)	0.78 (0.58–0.97)
Secondary or above	103 (69.6)	45 (30.4)	1	1
Residence				
Urban	214 (65.4)	113 (34.6)	1	1
Rural	131 (49.4)	134 (50.6)	1.67 (1.49–1.88)	−0.33 (−0.44 to −0.23)
Marital status				
Single	128 (66.7)	64 (33.3)	1	1
Married	182 (55.3)	147 (44.7)	1.07 (0.94–1.22)	0.08 (−0.11 to 0.28)
Widowed or divorced	35 (49.3)	36 (50.7)	1.70 (0.45–6.41)	0.46 (−0.43 to 1.35)
Religion				
Christian	239 (61.8)	148 (38.2)	1	1
Muslim	106 (51.7)	99 (48.3)	1.57 (1.29–1.92)	−1.77 (−4.36 to 0.81)
Perceived social support				
Poor	141 (57.8)	103 (42.2)	1	1
Moderate	103 (53.4)	90 (46.6)	1.19 (0.85–1.67)	0.36 (0.20–0.53)
Strong	101 (65.2)	54 (34.8)	0.73 (0.30–1.76)	0.35 (−0.16 to 0.85)
TB stigma score				
Low	105 (59.3)	72 (40.7)	1	1
Moderate	139 (59.9)	93 (40.1)	0.76 (0.50–1.15)	0.001 (−0.80 to 0.81)
High	101 (55.2)	82 (44.8)	0.73 (0.30–1.78)	−0.31 (−0.91 to 0.29)
Level of disability				
Low or no disability	121 (68.4)	56 (31.6)	1	1
Moderate disability	118 (51.1)	113 (48.9)	2.11 (1.03–4.29)	0.66 (−0.17 to 1.49)
Severe disability	106 (57.6)	78 (42.4)	1.52 (1.35–1.71)	1.86 (1.68–2.05)
Type of TB				
Pulmonary	211 (62.1)	129 (37.9)	1	1
Extra pulmonary	134 (53.2)	118 (46.8)	1.46 (1.07–2.00)	0.01 (−0.74 to 0.77)
HIV status				
Negative	260 (57.1)	195 (42.9)	1	1
Positive	42 (67.7)	20 (32.3)	0.81 (0.67–0.99)	−0.40 (−2.17 to 1.37)
Unknown	43 (57.3)	32 (42.7)	1.24 (0.59–2.61)	−0.87 (−1.10 to −0.65)
Annual household income				
Below median	174 (58.4)	124 (41.6)	1	1
Median or above	171 (58.2)	123 (41.8)	1.39 (1.15–1.67)	0.31 (0.24–0.38)
Alcohol risk				
Low	306 (59.1)	212 (40.9)	1	
Moderate- to-high	39 (52.7)	35 (47.3)	1.34 (1.14–1.57)	−1.18 (−3.98 to 1.62)
Khat risk				
Low	298 (60.1)	198 (39.9)	1	1
Moderate- to-high	47 (49.0)	49 (51.0)	1.39 (0.58–3.30)	1.12 (0.92–1.31)
Microorganisms perceived as cause of TB				
No	296 (58.0)	214 (42.0)	1	1
Yes	49 (59.8)	33 (40.2)	1.32 (0.37–4.71)	−0.27 (−0.91 to 0.37)
Perceived barriers to modern care				
No	264 (57.6)	194 (42.4)	1	
Yes	81 (60.5)	53 (39.5)	0.97 (0.77–1.21)	−0.001 (−0.87 to 0.87)
Perceived severity of TB				
Mild to moderate	89 (60.5)	58 (39.5)	1	1
Severe	256 (57.5)	189 (42.5)	0.95 (0.79–1.14)	−0.93 (−1.21 to −0.65)

AOR, adjusted odds ratio.

## Discussion

None of our hypotheses was supported: co-morbid depression was not associated with either diagnostic delay or indirect pathways to TB care, and depression did not appear to be a mediator of associations between various socio-demographic and clinical factors and diagnostic delay or pathways to TB care.

There were no previous studies to compare our findings. Two further factors require investigation before we accept the null hypothesises of no difference. First, only 71.0% of all estimated new TB cases in Ethiopia are detected (World Health Organization, 2016). We do not know whether the undetected 29.0% of cases are different from 71.0% in terms of depression status. Second, health extension workers in the Ethiopian health care system encourage people with TB symptoms to receive early diagnosis and treatment by going from home-to-home (Workie & Ramana, 2013). That means the role of depression in influencing diagnosis delay may be reduced. Health extension workers are more likely to help people with TB symptoms when the symptoms are clear. For example, we found that people with pulmonary TB had shorter diagnosis delay compared to those with extra-pulmonary TB where symptoms vary depending on the site (Pallangyo, 2001).

Our findings suggest that depression may not have any impact upon pathways to care for TB and the knowledge of microorganisms as a cause for TB may not be associated with where to go upon experiencing symptoms. These findings generally agree with previous studies. In Ethiopia, the choice of a healer for an illness largely depends on the reputation of the healer in their community (Jakonsson, 1991) rather than the symptoms felt or the perceived cause of the illness (Keegstra, 1986). When people fail with one, they go to another (Kloos *et al*., 1997).

Our findings that indirect pathway to TB care leads to diagnostic delay agrees with the existing evidence (Barker *et al*., 2006; Finnie *et al*., 2011). With all other socio-demographic variables in this study kept constant, better income was found to lead to diagnostic delay both directly and indirectly by leading to indirect pathways to TB care. Perceived good social support also contributed to diagnosis delay through an increasing visit to the traditional health care system. The explanation why people with better income and support encounter a longer delay before diagnosis was not clear. There is some evidence that traditional care is much more expensive than the modern one (Makanjuola, 2003). People with more resources may be exploring more options of treatments for their symptoms before accessing modern care. In Ethiopia, modern TB care is totally free.

Our findings that the absence of formal education, being a rural resident, having extra pulmonary TB, and alcohol use are associated with diagnostic delay agree with previous reports (Storla *et al*., 2008). Contrary to previous reports (Storla *et al*., 2008; Virenfeldt *et al*., 2014), HIV decreased diagnostic delay in our study probably because people who have known HIV status seek help more actively than those without HIV; they are people who have already sought help for HIV and been informed about potential symptoms of TB. The associations between gender, age, religion, and residence and diagnosis delay and pathways to TB care indicate the need for exploring the underlying cultural drivers (Bailey, 1987). The association between disability and diagnosis delay may show that people with a long period with illness develop severe forms of disability or people with disability find it harder to access services (Virenfeldt *et al*., 2014).

## Limitations

We measured depression using a screening tool. We also overly summarized pathways to care in to direct and indirect without considering back and forth visits or simultaneous use of both the traditional and conventional health systems. People may not remember exactly when the symptoms of TB started and, therefore, our estimates of diagnosis delay may not be accurate. Our findings are generalisable only to people with TB who were eventually attending the conventional care regardless of their pathways; specifically, it cannot be generalisable to those attending care in the traditional care system only. Eighty-five per cent of the sample reported to earn <1$/day/person (Ambaw *et al*., 2017), and higher income and social support are, therefore, only relative. Although there might be a tendency to under report income, it might not seriously affect regression outputs as those with higher income are likely to report higher values than those with lower income forming a pattern similar to the correct value. The other limitation was the absence of qualitative evidence on how each of the socio-demographic factors was connected to pathways to care and diagnosis delay.

## Conclusions

People with TB who had co-morbid depression visited the modern health care system as directly as and as soon as people with TB who did not have co-morbid depression. Depression was not mediating the relationship between diagnostic delay and factors associated with diagnostic delay. Co-morbid depression in people with TB could be diagnosed and treated at TB clinics by integrating mental health components into the TB management protocol. This recommendation can be used in other settings as well. How socio-demographic factors influence pathways to care and diagnosis delay require qualitative exploration.

## Supplementary material

For supplementary material accompanying this paper visit http://dx.doi.org/10.1017/gmh.2019.17. click here to view supplementary material
