# Supplementary figures and images for: Is depression associated with pathways to care and diagnosis delay in people with tuberculosis in Ethiopia?
Source: Glob Ment Health (Camb). 2019 Aug 23;6:e20. doi: 10.1017/gmh.2019.17 (PMC6737587; doi:10.1017/gmh.2019.17)

**
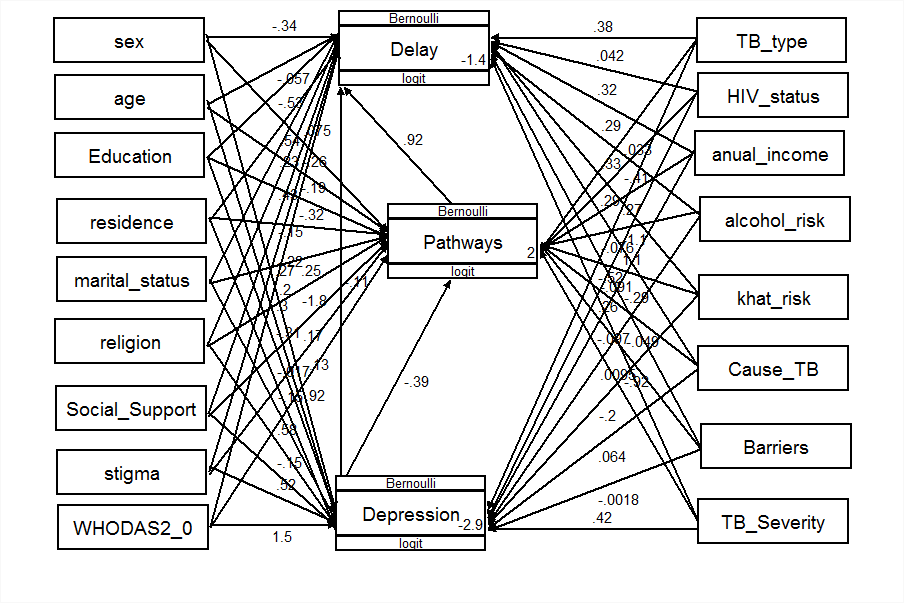
**

**Figure 1**: Path diagram of factors leading to diagnosis delay of TB

Supplement: Supplementary file 1 [file S2054425119000177sup001.docx]
